# Cutaneous TRPV4 Channels Activate Warmth-Defense Responses in Young and Adult Birds

**DOI:** 10.3389/fphys.2022.892828

**Published:** 2022-07-15

**Authors:** Caroline Cristina-Silva, Lara Amaral-Silva, Kassia Moreira Santos, Gabriela Monteiro Correa, Welex Candido da Silva, Marcia H. M. R. Fernandes, Glauber S. F. da Silva, Luciane H. Gargaglioni, Maria C. Almeida, Kenia C. Bicego

**Affiliations:** ^1^ Department of Animal Morphology and Physiology, Faculty of Agricultural and Veterinary Sciences, Sao Paulo State University, Jaboticabal, Brazil; ^2^ Department of Animal Science, Faculty of Agricultural and Veterinary Sciences, Sao Paulo State University, Jaboticabal, Brazil; ^3^ Institute of Biological Sciences, Department of Physiology and Biophysics, Federal University of Minas Gerais (ICB/UFMG), Belo Horizonte, Brazil; ^4^ Center for Natural and Human Sciences, Federal University of ABC (UFABC), São Bernardo do Campo, Brazil

**Keywords:** peripheral thermoreceptor, chicken, thermoregulation, Metabolism, body temperature, thermolysis, huddling

## Abstract

Transient receptor potential vanilloid 4 (TRPV4) channels are sensitive to warm ambient temperatures (T_a_s), triggering heat loss responses in adult rats in a T_a_s range of ∼26–30°C. In birds, however, the thermoregulatory role of TRPV4 has never been shown. Here, we hypothesized that stimulation of TRPV4 induces thermolytic responses for body temperature (T_b_) maintenance in birds, and that this function is already present in early life, when the T_a_ range for TRPV4 activation does not represent a warm condition for these animals. We first demonstrated the presence of TRPV4 in the dorsal and ventral skin of chickens (*Gallus gallus domesticus*) by immunohistochemistry. Then, we evaluated the effects of the TRPV4 agonist, RN1747, and the TRPV4 antagonists, HC067047 and GSK2193874, on T_b_ and thermoeffectors at different T_a_s in 5-day-old chicks and 60-day-old adult chickens. For the chicks, RN1747 transiently reduced T_b_ both in thermoneutrality (31°C) and in a cold T_a_ for this phase (26°C), which relied on huddling behavior inhibition. The TRPV4 antagonists alone did not affect T_b_ or thermoeffectors but blocked the T_b_ decrease and huddling inhibition promoted by RN1747. For the adults, TRPV4 antagonism increased T_b_ when animals were exposed to 28°C (suprathermoneutral condition for adults), but not to 19°C. In contrast, RN1747 decreased T_b_ by reducing metabolic rate and activating thermal tachypnea at 19°C, a T_a_ below the activation range of TRPV4. Our results indicate that peripheral TRPV4 receptors are functional in early life, but may be inhibited at that time when the range of activation (∼26–30°C) represents cold T_a_ for chicks, and become physiologically relevant for T_b_ maintenance when the activation T_a_ range for TRPV4 becomes suprathermoneutral for adult chickens.

## 1 Introduction

The maintenance of body temperature (T_b_) in an endotherm exposed to ambient temperature (T_a_) variation depends on the activity of neural circuits, which detect T_a_ in the peripheral thermoreceptors, integrate the thermal information in the brain, and recruit appropriate thermoeffectors for heat gain or heat loss (Bicego et al., 2007; Morrison, 2016). The thermosensitive transient receptor potential channels, called thermo-TRPs, emerged two decades ago as putative molecules involved in thermosensitivity (Patapoutian et al., 2003; Wang and Siemens, 2015). They are located on the cell membrane of a variety of cell types, such as neurons and epithelial cells, and have six transmembrane domains. *In vitro* studies of thermo-TRPs in mammals have shown that TRPV1, TRPV2, and TRPM3 are activated by noxious heat, TRPA1 is activated by noxious cold, TRPM8 and TRPC5 are activated by innocuous cold, and TRPM2, TRPM4, TRPM5, TRPV3, and TRPV4 are activated by moderate heat (Guler et al., 2002; Clapham, 2003; Patapoutian et al., 2003). Functional evidence (*in vivo*) of a thermoregulatory role for thermo-TRPs, however, is available for only a few channels, including TRPM8, TRPV3 and TRPV4, in rodents (Peier et al., 2002; Almeida et al., 2012; Vizin et al., 2015; Wang and Siemens, 2015; Scarpellini et al., 2019). *In vitro,* TRPV4 channels are specifically stimulated at temperatures of 27–34°C (Guler et al., 2002; Watanabe et al., 2002; Kauer and Gibson, 2009)*.* At least in rats they are expressed peripherally in the keratinocytes (Guler et al., 2002). *In vivo*, we have shown that peripheral TRPV4 receptors activate warmth-defense responses when adult rats are exposed to a range of T_a_s between ∼26 and 30°C (Vizin et al., 2015). Besides that, hypothalamic TRPV4 channels, which seems to be modulated by neurochemical signaling instead of changes in temperature, are also important for thermoregulatory responses to warm conditions in adult rats (Vizin et al., 2015; Scarpellini et al., 2019).

TRP channels are well preserved throughout evolution, and a phylogenetic analysis in birds, a vertebrate group that seems to have evolved endothermy following a different pathway from mammals (Legendre and Davesne, 2020), indicates that they have a copy of each gene coding for TRPs: V1, V2, V3, V4, V6, M2, M5, M8, and A1 (Saito and Shingai, 2006). Some studies *in vitro* show that similarly to mammals, chicken TRPM8 is activated in mild cold and by menthol (Chuang et al., 2004; Myers et al., 2009), whereas chicken TRPA1 is activated by noxious heat, contrary to what is found in mammals (Saito et al., 2014). Additionally, chicken TRPV1 also senses noxious heat (Jordt and Julius, 2002) and seems to be involved in the T_b_ drop during endotoxemic shock in chicks (Nikami et al., 2008). TRPV4 channels were described in the rictus associated with Herbst corpuscle mechanoreceptors in pigeons (Cabo et al., 2013). However, as far as we know there is no study showing the presence of any TRP channels in the skin or exploring their role in avian thermoregulation (*in vivo*).

In mammals and birds, along with development and growth, the maturation of thermogenesis and increase in insulation make the thermal comfort and thermoneutral zone shift overtime from warmer to colder T_a_s (Alberts, 1978; Nichelmann and Tzschentke, 2002; Mortola and Labbe, 2005). As a consequence, changes in thermoregulation are observed during the animal’s development, as thermosensitivity changes with aging, i.e., a T_a_ that is considered neutral in early life, is normally supraneutral for adults. In this way, precocial birds are excellent models for studying the development of thermogenesis since endothermy is already present as soon as they hatch and the shift in thermosensitivity can be observed by activation of heat loss/heat conservation thermoeffectors in early life (Mathiu et al., 1991; Khandoker et al., 2004; Mortola and Maskrey, 2011; Toro-Velasquez et al., 2014; Cristina-Silva et al., 2021; Amaral-Silva et al., 2022).

In the present study, we tested the hypothesis that TRPV4 stimulation induces thermolytic responses for T_b_ maintenance in birds at warm T_a_s, and that this function is already present in early life, when the TRPV4 activation T_a_ range does not represent a suprathermoneutral condition. First, immunohistochemistry was used to detect TRPV4 channels in the dorsal and ventral skin of 60-day-old adult chickens. To assess the role of TRPV4 channels in thermoregulation *in vivo*, we treated 5-day-old chicks and 60-day-old chickens with a selective agonist (RN1747) and antagonists (HC067047 and GSK 2193874) of TRPV4 (Vincent et al., 2009; Everaerts et al., 2010). The effects of the TRPV4 agonist and antagonists were evaluated on T_b_ and the thermoeffectors, such as oxygen consumption (index of thermogenesis), pulmonary ventilation (index of thermal tachypnoea in heat) and huddling behavior (heat conservation mechanism in chicks) in different T_a_s.

## 2 Materials and Methods

### 2.1 Animals

#### 2.1.1 Five-Day-Old Chicks

Hatchlings from *Gallus gallus domesticus*, of the Carijo lineage (Plymouth rock), were locally supplied (Globoaves, Itirapina, SP, Brazil) and raised at the Department of Animal Morphology and Physiology (FCAV-UNESP). The chicks were kept in climatic chambers (Premium Ecologica, Belo Horizonte, MG, Brazil) at 31–32°C (age-appropriate) with a light:dark cycle of 14:10 h (lights on at 6 a.m.) (Amaral-Silva et al., 2020, Amaral-Silva et al., 2021; Cristina-Silva, 2021). The experiments were conducted in 5-day-old chicks (60–70 g), a phase when thermogenesis is considered to be fully established (Nichelmann and Tzschentke, 2002; Tazawa et al., 2004; Amaral-Silva et al., 2020).

#### 2.1.2 Sixty-Day-Old Chickens

A separate group of Carijo hatchlings (Globoaves, Itirapina, SP, Brazil) were housed in climatic chambers of the FCAV-UNESP-Jaboticabal poultry sector, and raised at an appropriate T_a_ for each stage of development (first week, 31–32°C; second week, 25–28°C; third week, 20–22°C; forth through eighth weeks, 19–20°C; De Oliveira et al., 2006). A light:dark cycle of 14:10 h was kept the entire time. The experiments were then conducted when the birds achieved 55–65 days (2.5–3.5 kg), an age when birds were considered adults for this lineage.

All animals were provided standard food and water *ad libitum*. The experiments were carried out between 8:00 a.m. and 5:00 p.m. to avoid any influence of the daily T_b_ cycle. All procedures were conducted according to the guidelines of the National Animal Experimentation Control Council (CONCEA-Brazil) and with the approval of the local Animal Care and Use Committee (CEUA-FCAV-UNESP-Jaboticabal).

### 2.2 Immunohistochemistry of the Dorsal and Ventral Skin

After the end of the experiments, 60-day-old adult chickens were deeply anesthetized and skin from the dorsal and ventral regions of the body was sampled, allocated in embedding cassettes and immediately immersed in 4% paraformaldehyde solution (PFA, Sigma-Aldrich Brazil Ltda. Sao Paulo, SP, Brazil) for 2 h. In sequence, the cassettes containing the skin were immersed in 70% alcohol solution for at least 2 days. The skin samples were then embedded in paraffin and the blocks were sliced in 40-µm transversal sections using a microtome (Leica RM2255, Wetzlar, HE, Germany).

For immunohistochemistry, the sections were deparaffinized and incubated for 30 min in an antigen retrieval solution (Dako, Glostrup, Denmark) at 70°C. Subsequently, the slices were washed in 1.5% hydrogen peroxide for 30 min, and were then incubated in 3% horse serum (Sigma-Aldrich Brazil Ltda. Sao Paulo, SP, Brazil) for 1 h at room temperature to prevent non-specific binding. The slices were incubated at 25°C for 24 h with a rabbit anti-TRPV4 primary antibody (Abcam 39260; dilution 1:200; Cambridge, United Kingdom), followed by a 4-h incubation in darkness with a fluorescent anti-rabbit secondary antibody (Alexa Fluor 488; dilution 1:500; Thermo Fisher Scientific Inc. Waltham, MA, United States). Finally, the sections were placed on gelatin-coated slides. After drying, they were covered by coverslips glued with a specific fluorescence preservative (ProLong Gold Antifade Reagent, Invitrogen, Carlsbad, CA, EUA) and observed under a microscope (Axio Imager Z2; Carl Zeiss do Brasil Ltda. Sao Paulo, Brazil) at a wavelength of 488 nm. A negative control was prepared following the same steps, except for the absence of the primary antibody.

### 2.3 Body Temperature (T_b_)

Chicks, free to walk around the brooder (Protocols 1 and 2, described below), had their T_b_ measured by a colonic sensor (Yellow spring Instrument, Co., Ohio, United States). The sensor was inserted 3 cm through the cloaca into the colon and was connected to a tele-thermometer (45TUC, Yellow spring Instrument Co., Ohio, United States). To avoid the influence of stress on T_b_ due to manipulation on the day of the experiment, the chicks were previously trained to the procedure from day 2 to day 4 (3 measurements/day, 1/hour). Chicks in the respirometry chamber (Protocol 4, described below) had their T_b_ monitored by telemetry using a temperature-sensitive tag (Biomark HPR Plus Reader, Boise, ID, United States) implanted in the coelomic cavity. For this, chicks were anesthetized with isoflurane (5% for induction and 1% for maintenance in pure O_2_, using a face mask). The tag was then inserted through the skin and abdominal muscle using an application needle (AnimalTAG, Sao Carlos, SP, Brazil). The small incision point was closed using surgical glue (Dermabond Topical Skin Adhesive; Johnson & Johnson, Sao Paulo, SP, Brazil). Antibiotic (enrofloxacin, intramuscular; 10 mg kg^−1^; Bayer SA, Sao Paulo, SP, Brazil) and analgesic anti-inflammatory (flunixin meglumine, intramuscular; 2.5 mg kg^−1^; MSD Saude Animal, Sao Paulo, SP, Brazil) agents were prophylactically administered, and the chick stayed in observation until recovery. The entire procedure lasted about 10 min. The sensor was implanted 2–3 days before the experiments. At the day of the experiment, individual T_b_s were recorded in real time by telemetry using a Biotherm reader (Biomark HPR Plus Reader, Boise, ID, United States) and uploaded to a computer (BioTerm software). Both sensors (colonic and abdominal) were calibrated against a certified thermometer (0.1°C precision). The T_b_s measured by the colonic and abdominal sensors were highly correlated (R^2^ = 0.9782).

The T_b_ of adult animals (Protocols 5 and 6 described below) was monitored by temperature dataloggers (SubCue, Calgary, AB, Canada) implanted in the coelomic cavity via median laparotomy. To this end, animals were anesthetized using isoflurane (5% for induction using a face mask followed by intubation and maintenance with 1% in pure O_2_). A small incision in the skin and muscle layers at the ventral midline, just caudal to the breast muscle, allowed for the insertion of the sensor into the coelomic cavity. Subsequently, the abdominal muscles and skin were sutured in layers, the isoflurane flow was closed, and we waited until animal itself removed the anesthesia tube from the trachea by reflex. The chickens were prophylactically treated with antibiotic (enrofloxacin, intramuscular; 10 mg kg^−1^; Bayer SA, Sao Paulo, SP, Brazil) and with analgesic anti-inflammatory (flunixin meglumine, intramuscular; 2.5 mg kg^−1^; MSD Saude Animal, Sao Paulo, SP, Brazil) agents. The surgery was performed at least 2 days before the experiment. At the end of the experiments, chickens were deeply anesthetized with 2,2,2-tribromoethanol (250 mg kg^−1^; Sigma-Aldrich Brasil Ltda. Sao Paulo, SP, Brazil) to remove the sensors. The data stored in the sensors was uploaded to the computer (Subcue software, Calgary, AB, Canada) and calibrated according to the manufacturer’s recommendations.

### 2.4 Oxygen Consumption

Oxygen consumption ( $\dot{V}$ O_2_) was measured using an open-flow respirometry system. One animal at a time was placed inside a respirometer (3 L for chicks and 40 L for adult chickens) and continuous gas flow (room air) was maintained through the chamber at 1 L min^−1^ for chicks and 5 L min^−1^ for adult chickens using a mass flow system with flowmeters (MSF and FK-100; Sable Systems, Las Vegas, NV, United States). The temperature within the respirometer was set according to the protocol described below. A subsample of the outflow air was pulled (180 ml min^−1^; SS4; Sable Systems, Las Vegas, NV, United States), passed through a water vapor pressure analyzer (RH300; Sable Systems, Las Vegas, NV, United States), dried (Drierite, with indicator, eight mesh, Sigma-Aldrich Brazil Ltda. Sao Paulo, SP, Brazil), and finally pulled into an O_2_ analyzer (PA-10; Sable Systems, Las Vegas, NV, United States). Water vapor pressure (WVP; kPa) and barometric pressure (kPa) were later used to correct the flow. The analyzer and flowmeters were connected in line with an analog-to-digital converter (PowerLab; ADInstruments, Sydney, NSW, Australia), and data were recorded using LabChart (ADInstruments, Sydney, NSW, Australia). The analyzers were calibrated before each experiment using nitrogen as zero and dry ambient air as 20.95% oxygen. As CO_2_ was neither analyzed nor scrubbed, the $\dot{V}$ _E_O_2_ was calculated using the following equation (Koteja, 1996): $\dot{V}$ _E_O_2_ = [FRe (FiO_2_—FeO_2_)]/[1—FiO_2_ (1—RQ)], where FRe is the excurrent flow rate, FiO_2_ is the incurrent fractional concentration of oxygen (baseline), FeO_2_ is the excurrent fractional concentration of oxygen, and RQ is the respiratory quotient (considered to be 0.85). Data are shown in STPD (standard conditions of temperature, pressure and dry air).

### 2.5 Pulmonary Ventilation

Pulmonary ventilation ( $\dot{V}$ _E_) was measured concurrently with $\dot{V}$ _E_O_2_ using a FD141 pressure transducer-based spirometer (ADInstruments, Sydney, NSW, Australia) connected to the respirometer. Breathing frequency (f) was calculated by counting the pressure peaks per time. Tidal volume (V_T_) was calculated using the following formula (Drorbaugh and Fenn, 1955): V_T_ = A (V_cal_/P_cal_) [T_b_ (P_B_ -Pc_H2O_)]/{[T_b_ (P_B_—Pc_H2O_)]—[T_ch_ (P_B_—Pb_H2O_)]}, where V_T_ is the tidal volume, A is the wave amplitude, V_cal_ is the calibration volume, P_cal_ is the calibration pressure, T_b_ is the body temperature, P_B_ is barometric pressure, Pc_H2O_ is the water vapor pressure in the chamber, T_ch_ is the temperature inside the respirometry chamber, and Pb_H2O_ is the water vapor pressure of the air inside the animal’s body. The system was calibrated for volume by comparison with the pressure produced by known volumes of air injected into the system with a syringe. Finally, pulmonary ventilation was calculated as $\dot{V}$ _E_ = f × V_T_.

### 2.6 Drugs

The selective TRPV4 antagonist, HC-067047 {2-Methyl-1-[3-(4-morpholinyl) propyl]-5-phenyl-N-[3-(trifluoromethyl)phenyl]-1H-pyrrole-3-carboxamide} (Everaerts et al., 2010; Shibasaki et al., 2015), was purchased from Tocris Bioscience (Bristol, United Kingdom). It was dissolved in a solution of 10% ethanol in sterile saline, and the doses of 10, 50, and 100 μg kg^−1^ were chosen based on results we previously presented in rats (Vizin et al., 2015) and on pilot experiments using chickens. The selective TRPV4 antagonist, GSK 2193874 [3-(1,4′-Bipiperidin)-1′-ylmethyl]-7-bromo-N-(1-phenylcyclopropyl)-2-[3-(trifluoromethyl)phenyl]-4-quinolinecarboxamide; Cheung et al., 2017), was purchased from Sigma-Aldrich Brazil Ltda (Sao Paulo, SP, Brazil). It was dissolved in a solution of 1% DMSO in sterile saline, and the doses of 100 and 500 μg kg^−1^ were chosen based on our previous study (Scarpellini et al., 2019). Both antagonists were applied intramuscularly (i.m.) in the leg (Vizin et al., 2015).

The selective TRPV4 agonist, RN1747 [1-(4-Chloro-2-nitrophenyl) sulfonyl-4-benzylpiperazine, Tocris Bioscience, Bristol, United Kingdom], was dissolved in propylene glycol at concentrations of 0.2, 0.5, and 1.0 mg ml^−1^ and applied topically to the skin for activation of cutaneous TRPV4 channels. Doses of 0.75, 1.87, and 3.75 mg kg^−1^ were applied to the dorsal skin in chicks and to the dorsal and ventral skin in adults. The liquid was carefully applied with the help of a needleless syringe and it was well spread on the animal’s surface with circular movements. Because of its viscous consistency, the solution did not flow out of the applied area. For the chicks, we applied ∼0.3 ml of the RN1747 solution and for the adults, a volume of ∼11.25 ml, with half of the volume applied to each portion of the skin (dorsal or ventral) (total of application in both ages: 3.75 ml kg^−1^). The choice of doses and application method were based on a previous study (Vizin et al., 2015).

### 2.7 Protocols

Protocols 1-4 were performed in 5-day-old Carijo chicks, and Protocols five and six were performed in 60-day-old adult Carijo chickens. To investigate the effects of TRPV4 activation on thermoeffectors, we combined pharmacological blockage of TRPV4 (using specific antagonists) with both chemical (topical application of RN1747 in chicks and adults) and thermal (exposure of adults to 28°C) activation of the channel. The animals were kept at 30–31°C for at least 60 min in order to promote skin vasodilation and deliver the circulating antagonist (i.m. injected) to its presumed site of action (skin receptors) before being subjected to the thermal stimulus (26°C for chicks; 28 and 19°C for adults), as previously described in rats (Almeida et al., 2012; Vizin et al., 2015). The temperatures of 31 and 26°C were chosen for Protocols 1–4 because 31°C is thermoneutral for five- to 8-day-old chicks (Coleone et al., 2009; Amaral-Silva et al., 2021, 2022; Cristina-Silva et al., 2021) and 26°C is in the range of TRPV4 activation *in vivo*, but is considered to be cold for chicks (Vizin et al., 2015; Amaral-Silva et al., 2021, 2022; Cristina-Silva et al., 2021). Different animals were used for each protocol, and they were deeply anesthetized and killed immediately after the end of the experiments.

#### 2.7.1 Protocol 1: Effect of Chemical Activation and Inhibition of the TRPV4 Channels on T_2_ of 5-day-Old Chicks at 31°C and 26°C

Chicks received: 1) topical application of the TRPV4 agonist, RN1747 (0.2, 0.5 or 1.0 mg ml^−1^), or its vehicle (100% propylene glycol; 3.75 ml kg^−1^) on the dorsal skin; or 2) intramuscular (i.m.) injection of the TRPV4 antagonist, HC067047 (10, 50 or 100 μg kg^−1^), or its vehicle (90% saline +10% ethanol; 1 ml kg^−1^). Colonic temperature was measured just before and hourly up to 240 min after each treatment.

#### 2.7.2 Protocol 2: Effect of the TRPV4 Antagonist on T_2_ of 5-Day-Old Chicks at 31°C and 26°C Following Chemical Activation of TRPV4

Chicks were injected with 50 μg kg^−1^ (i.m.) of HC067047 or its vehicle (1 ml kg^−1^). One hour later, they were topically applied with RN1747 (0.5 mg ml^−1^) or its vehicle (3.75 ml kg^−1^). This generated four treatment combinations: 1) vehicle of HC067047 + vehicle of RN1747 (sham); 2) HC067047 + vehicle of RN1747 (antagonist effect only); 3) vehicle of HC067047 + RN1747 (agonist effect only); 4) HC067047 + RN1747 (inhibition of TRPV4 chemical activation). Colonic temperature was measured before the first treatment and hourly for five consecutive hours in all groups. The antagonist and agonist doses were chosen based on the results of the Protocol 1. The experiment was conducted: 1) at a neutral T_a_ of 31°C during the whole period of T_b_ measurements; and 2) started at 31°C, which was maintained for 1 h after the antagonist injection (to facilitate access of the circulating drug to the skin, as explained above), and at the moment of the agonist topical application, the T_a_ was reduced to 26°C.

#### 2.7.3 Protocol 3: Effects of the TRPV4 Antagonist on Huddling Behavior of 5-Day-Old Chicks Exposed to 26°C Following Chemical TRPV4 Activation

One day before the experiments, chicks were separated into groups of five individuals and kept in climatic chambers at 31°C. On the following day, chicks were injected with HC067047 (50 μg kg^−1^) or vehicle (90% saline +10% ethanol; 1 ml kg^−1^) and maintained at 31°C for 60 min. Then, they were topically applied with RN1747 (0.5 mg ml^−1^) or its vehicle (100% propylene glycol; 3.75 ml kg^−1^) and exposed to 26°C for the manifestation of cold-induced huddling behavior. The treatment combinations presented in Protocol two were repeated in this protocol (vehicle of HC067047 + vehicle of RN1747; HC067047 + vehicle of RN1747; vehicle of HC067047 + RN1747; and HC067047 + RN1747). Chicks were monitored using a webcam (LifeCam HD-3000, Microsoft, Redmond, WA, EUA) fixed about 1 m above the experimental chambers and programmed to take pictures with a 2-min interval from the time of pretreatment up to about 270 min after the topical treatment. The number of single chicks (ungrouped animals) was counted, and the area occupied by the five chicks in the group was measured using ImageJ (FIJI) to infer huddling behavior for heat conservation (Dantonio et al., 2015).

#### 2.7.4 Protocol 4: Effects of the TRPV4 Antagonist on the Effect of Chemical TRPV4 Activation on T_b_, Oxygen Consumption ( $\dot{V}$ O_2_) and Ventilation ( $\dot{V}$ _E_) of 5-Day-Old Chicks at 31°C

The selective TRPV4 antagonist, GSK2193874, was used in this protocol (Cheung et al., 2017; Scarpellini et al., 2019) to confirm the TRPV4 inhibition effect on the T_b_ of chicks and to verify the metabolic thermoeffectors affected. Resembling Protocols 2 and 3, chicks were pretreated with 50 μg kg^−1^ (i.m.) of the TRPV4 antagonist, GSK2193874, or its vehicle (1% DMSO; 1 ml kg^−1^), and after 60 min, were topically applied with 0.5 mg ml^−1^ of the TRPV4 agonist, RN1747, or its vehicle (100% propylene glycol; 3.75 ml kg^−1^). This also generated four treatment combinations: 1) vehicle of GSK2193874 + vehicle of RN1747 (sham); 2) GSK2193874 + vehicle of RN1747 (antagonist effect only); 3) vehicle of GSK2193874 + RN1747 (agonist effect only); 4) GSK2193874 + RN1747 (inhibition of TRPV4 chemical activation). The chicks in this protocol were allocated for 30 min in the respirometer for habituation. After that, oxygen fraction was recorded continuously from 20 min before the pretreatment until 240 min after the topical treatment. Ventilation was recorded during 2 min every 20 min, when the respirometer was closed for baseline recordings (FiO_2_). $\dot{V}$ O_2,_ V_T_ and *f* were determined for each 20-min interval. T_b_ was recorded every 5 min. T_a_ in the respirometer was monitored in real time by thermopar sensors (ADInstruments, Sydney, NSW, Australia) and controlled inside a climatic chamber (BOD, FANEM, Sao Paulo, SP, Brazil).

#### 2.7.5 Protocol 5: Effects of Chemical Inhibition of TRPV4 on T_b_, $\dot{V}$ O_2_ and $\dot{V}$ _2_ in Adult Chickens Exposed to 28°C and 19°C

Sixty-day-old chickens, previously implanted with a T_b_ sensor, were exposed to 30°C for 50 min before the experiments (to facilitate the access of the circulating drug to the skin, as explained above). T_b_ was measured during this phase. After that, this protocol was conducted in two different ways: 1) animals were injected with 100 or 500 μg kg^−1^ of the TRPV4 antagonist, GSK2193874, or its vehicle (1% DMSO; 1 ml kg^−1^) and transferred to a respirometer at 28°C (warm for this age) for recordings of T_b_, $\dot{V}$ O_2,_ V_T_ and f for 180 min; or 2) animals were injected with 500 μg kg^−1^ of the TRPV4 antagonist, GSK2193874, or its vehicle (1% DMSO; 1 ml kg^−1^) and transferred to a respirometer at 19°C (mild cold for this age) for recordings of T_b_, $\dot{V}$ O_2,_ V_T_ and f for 180 min. The respirometer was placed inside a climatic chamber (BOD, FANEM, Sao Paulo, SP, Brazil) continuously programmed to meet T_a_s set according to the experimental protocol considering the internal temperature of the respirometer measured by thermopar sensors (ADInstruments, Sydney, NSW, Australia).

#### 2.7.6 Protocol 6: Effects of Chemical Activation of TRPV4 on T_b_, $\dot{V}$ O_2_ and $\dot{V}$ _E_ of Adult Chickens Exposed to 19°C

After the initial warming phase at 30°C (see Protocol 5), 60-day-old chickens were topically applied with 0.5 mg ml^−1^ of the TRPV4 agonist, RN1747, or its vehicle (100% propylene glycol; 3.75 ml kg^−1^) equally distributed on the ventral and dorsal skin. They were then transferred to a respirometer at 19°C for recordings of T_b_, $\dot{V}$ O_2,_ V_T_ and f for 180 min. Similar to Protocol 5, the respirometer was placed inside a climatic chamber (BOD, FANEM, Sao Paulo, SP, Brazil) and its internal temperature was precisely monitored in real time by thermopar sensors (ADInstruments, Sydney, NSW, Australia).

### 2.8 Data Processing and Analysis

All data are presented as mean ± standard error. The effects on the peak of delta T_b_ (calculated by subtracting the peak value of the treatments from the respective controls) of the animals that received different doses of the TRPV4 agonist and the antagonist were compared using one-way ANOVA. The effects of the TRPV4 agonist and antagonists on the variables evaluated in chicks (T_b_, $\dot{V}$ _E_O_2,_ $\dot{V}$ _E,_ V_T,_ and f, number of single chicks, and area occupied by a group) and adult chickens (T_b_, $\dot{V}$ _E_, O_2_, $\dot{V}$ _E_, V_T_, and f) were tested using repeated measures two-way ANOVA with treatment and time as factors. The differences among means were evaluated by the Sidak post-hoc test. Significant differences were declared at *p* < 0.05.

## 3 Results

### 3.1 Immunohistochemistry for TRPV4 in the Dorsal and Ventral Skin of Adult Chickens

**FIGURE 1:**
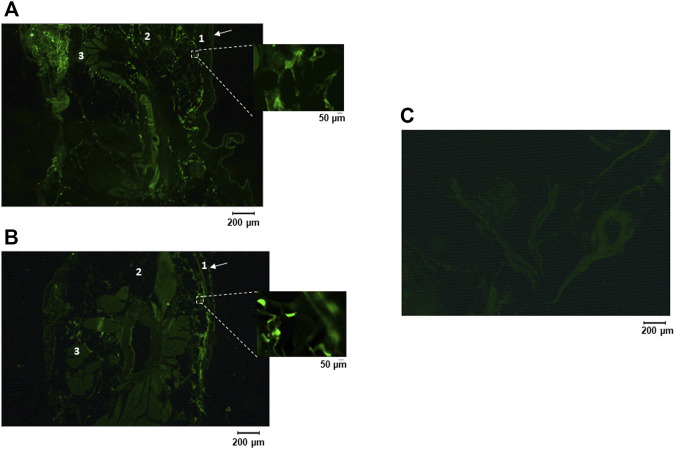
Representative photomicrographs of immunostaining for TRPV4 in the skin of adult chickens: ventral **(A)** and dorsal **(B)** cut of the skin; negative control without the primary antibody **(C)**. TRPV4 is seen as a green staining. Arrow: Stratum corneum; 1: Epidermis; 2: Dermis; 3: Muscle.

Demonstration of the presence of TRPV4 channels (green immunostaining) in the ventral and dorsal skin of chickens is shown in fluorescence photomicrographs (Figure 1A,B, respectively). TRPV4 was expressed in the layers of the dermis and subcutis both in the ventral and dorsal sites. A negative control is presented in Figure 1C to confirm the absence of unspecific staining without the primary antibody, anti-TRPV4.

### 3.2 Thermoregulatory Responses to Chemical Activation and Inhibition of TRPV4 Channels in 5-Day-Old Chicks

*Protocol 1—T*he topical application of the selective agonist for TRPV4, RN1747, significantly reduced the peak of delta T_b_ (at 120 min for all) in 5-day-old chicks when applied in concentrations of 0.5 and 1.0 mg ml^−1^, but not when applied at 0.2 mg ml^−1^ (Table 1). On the other hand, the selective TRPV4 antagonist, HC067047, alone did not affect the chicks’ T_b_ in the three doses applied, 10, 50 and 100 μg ml^−1^ (peak at 120 min; Table 1).

**TABLE 1:** Peak of delta body temperature (T_b_) of 5-day-old chicks under thermoneutral condition (T_a_ = 31°C) that received either topical administration (3.75 ml kg^−1^) of the TRPV4 agonist RN1747 (0.2, 0.5 and 1 mg ml^−1^) on the dorsal skin, or intramuscular (i.m.; 1 ml kg^−1^) injection of the TRPV4 antagonist HC067047 (10, 50 or 100 μg kg^−1^), compared to the respective vehicles (topical: propylene glycol; i.m.: 90% saline +10% ethanol).

	*Peak ± SEM (delta °C)*	*Peak diff* *^a^* (*delta °C*)	*p value*
***Vehicle*** (***100% propyleneglycol, topical***) (***12***)	−0.05 ± 0.05		
***RN1747, 0.2 mg ml*** ^***−1***^ ***topical (7)***	−0.05 ± 0.05	0	>0.999
***RN1747, 0.5 mg ml*** ^***−1***^ ***topical*** (***14***)	−0.30 ± 0.07	−0.25	0.0255^*^
***RN1747, 1.0 mg ml*** ^***−1***^ ***topical (7)***	−0.45 ± 0.07	−0.40	0.0022^*^
			F_(3,36)_ = 7.440
***Vehicle*** (***90% saline +10% etanol, i.m.***) (***11***)	0.30 ± 0.1		
***HC067047, 10 μg kg*** ^***−1***^ ***i.m.*** (***12***)	0.15 ± 0.08	−0.15	0.7503
***HC067047, 50 μg kg*** ^***−1***^ ***i.m.*** (***11***)	0.40 ± 0.05	0.10	0.9829
***HC067047, 100 μg kg*** ^***−1***^ ***i.m.*** (***11***)	0.40 ± 0.07	0.10	0.9038
			F_(3,41)_ = 2.118

Values are expressed as mean ± SEM., Number of animals in each group is shown in parentheses.

a Peak differences were calculated subtracting the peak value of the treatments (RN1747 and HC067047) from the respective controls (100% propylene glycol and 90% saline +10% ethanol) 2 hours after the applications at 31°C. *Significant *p* value for peak differences (one-way ANOVA; Sidak post-hoc test).

**FIGURE 2:**
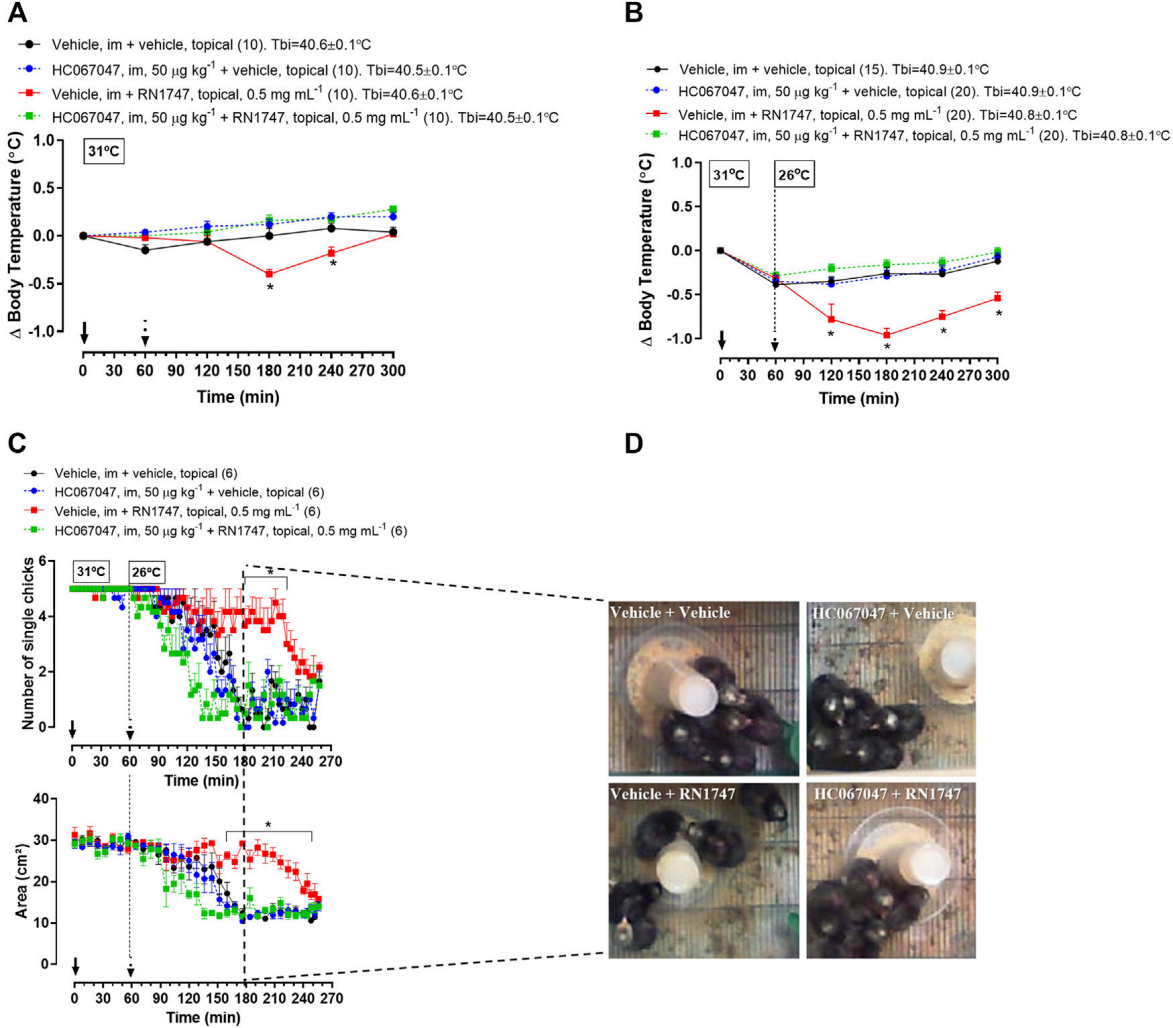
**(A,B)** Effect of pre-treatment with the TRPV4 antagonist HC067047 (intramuscular injection, i.m.; 50 μg kg^−1^; vehicle is 90% saline +10% ethanol; 1 ml kg^−1^) on the body temperature change induced by the TRPV4 agonist RN1747 (topical application on the dorsal skin; 0.5 mg ml^−1^; vehicle is 100% propyleneglycol; 3.75 ml kg^−1^) in 5-day-old chicks under thermoneutral condition (T_a_ = 31°C; **A**) or exposed to cold (T_a_ = 26°C; **B**). Tbi, initial body temperature. **C**) Influence of the pre-treatment with HC067047 on the effect of RN1747 on the huddling behavior induced by cold exposure (T_a_ = 26°C) in 5-day-old chicks, as assessed by the number of single chicks (top plot) and the area occupied by the group of five chicks (bottom plot). **D**) Representative images of each treatment showing the area occupied by the animals at the time 180 min from the graphs in **C** (2 hours after the agonist application). All values are expressed as mean ± SEM. The continuous arrow indicates the moment of the antagonist injection and the dashed arrow indicates the time of the topical application of the agonist. The number of animals in each group is shown in parentheses. *p < 0.05, significant difference from the vehicle group at the same time point (two-way ANOVA; Sidak post-hoc test).

*Protocol 2—*RN1747 (0.5 mg ml^−1^) caused a decrease in T_b_ from 120 to 180 min following application (Figure 2A; 180–240 min; treatment effect: *p* < 0.0001, F_(3, 36)_ = 10.42) when the chicks were in the thermoneutral condition. A more accentuated T_b_ decrease from 60 to 240 min following application of RN1747 (Figure 2B; 120–300 min; treatment effect: *p* < 0.001, F_(3, 71)_ = 16.79) was observed when chicks were exposed to cold (26°C—activation range of TRPV4). The antagonist injection alone had no effect on T_b_, which followed the same pattern as the control animals (vehicle i.m. + vehicle topical). However, the HC067047 effectiveness in inhibiting TRPV4 was demonstrated by the inhibition of the T_b_ decrease caused by the agonist, RN1747, at both thermoneutral and cold conditions (Figures 2A,B).

*Protocol 3—*The effect of the agonist and antagonist drugs on T_b_ was accompanied by the behavioral thermoregulation. Exposure to cold (26°C) induced huddling behavior in the 5-day-old chicks, which was observed by the reduction of the number of single chicks (Figure 2C; *p* < 0.001, F_(3, 20)_ = 47.65) and the area occupied by the group of chicks (Figure 2C; *p* < 0.001, F_(3, 20)_ = 59.08). Topical application of RN1747 inhibited the huddling behavior in response to cold, as it did not reduce the number of single chicks (Figure 2C; 170–220 min; *p* < 0.001, F_(3, 20)_ = 11.39) or the area occupied by the group of chicks (Figure 2D; 160–250 min; *p* < 0.001, F _(3, 20)_ = 50.21). Injection of HC067047 alone did not affect the chicks’ behavior, but its injection prevented the inhibition of huddling caused by the agonist, RN1747; thus, the chicks huddled in response to cold, similarly to the controls. The effects of cold and pharmacological activation/inhibition of TRPV4 in 5-day-old chicks’ behavior is exemplified in images in Figure 2D. It is possible to observe that the only group showing no huddling is the one that received the “vehicle + RN1747” treatment.

**FIGURE 3:**
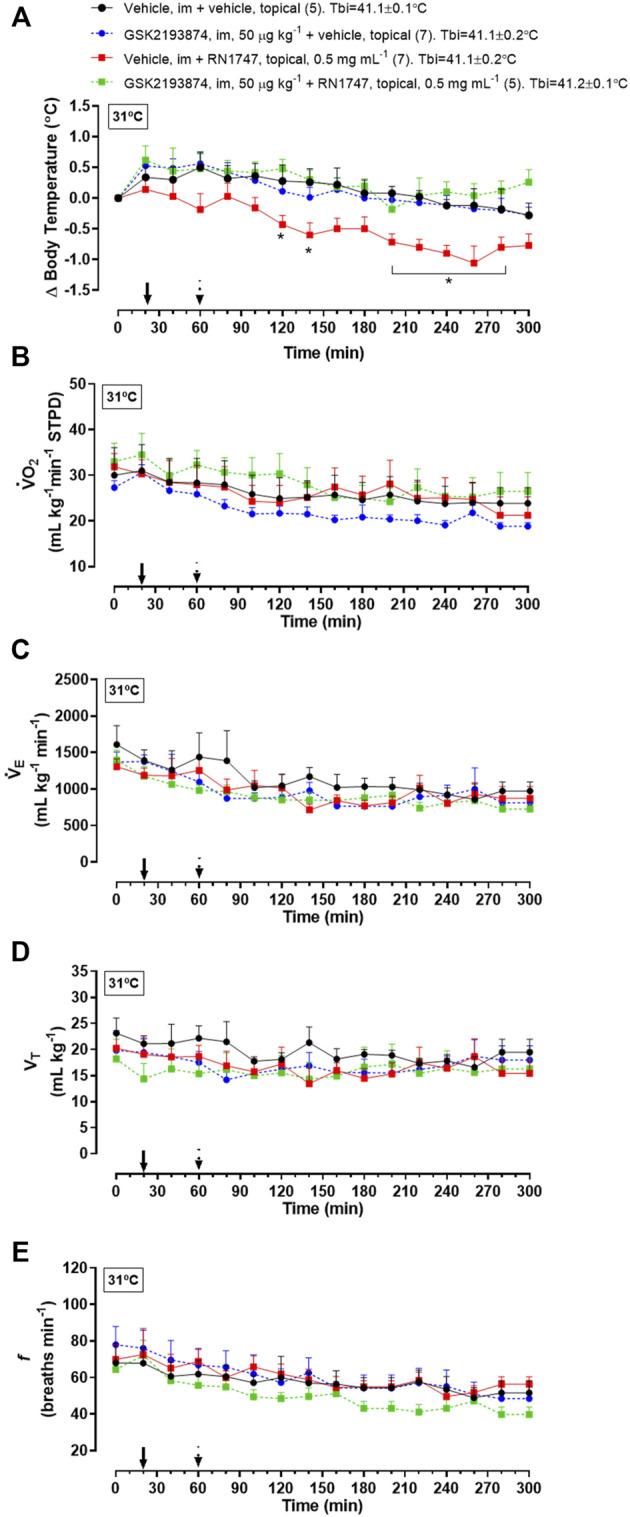
Effect of pre-treatment with the TRPV4 antagonist, GSK2193874 (intramuscular injection, i.m.; 50 μg kg^−1^; vehicle is DMSO 1%; 1  ml kg^−1^), on change in body temperature (delta; **A**), oxygen consumption ( $\dot{V}$ O_2_; **B**), ventilation ( $\dot{V}$ _E_; **C**), tidal volume (V_T_; **D**) and breathing frequency (*f*; **E**) induced by treatment with the TRPV4 agonist, RN1747 (topical application on the dorsal skin; 0.5 mg ml^−1^; vehicle is 100% propylene glycol; 3.75 ml kg^−1^), in 5-day-old chicks under thermoneutral condition (T_a_ = 31°C). T_b_i, initial body temperature. All values are expressed as mean ± SEM. The continuous arrow indicates the moment of the antagonist injection and the dashed arrow indicates the time of the topical application of the agonist. The number of animals in each group is shown in parentheses. *p < 0.05, significant difference from the vehicle group at the same time point (two-way ANOVA; Sidak post-hoc test).

*Protocol 4*—Under thermoneutrality, the selective TRPV4 antagonist, GSK2193874, had a similar effect on T_b_ compared to the antagonist, HC067047. By itself, it did not affect T_b_, but it prevented the T_b_ decrease caused by RN1747 (Figure 3A; 120–140 min; 200–280 min; treatment effect: *p* < 0.01, F_(3,21)_ = 5.40), and the chicks’ T_b_ was comparable to that of the controls. Despite the T_b_ changes, no difference in $\dot{V}$ O_2_ (Figure 3B; treatment effect: *p* = 0.66, F_(3,21)_ = 0.53), $\dot{V}$ _E_ (Figure 3C; treatment effect: *p* = 0.53, F_(3,21)_ = 0.76), V_T_ (Figure 3D; treatment effect: *p* = 0.69, F_(3,21)_ = 0.48), or *f* (Figure 3E; treatment effect: *p* = 0.58, F_(3,21)_ = 0.66) was observed among the treatments.

### 3.3 Thermoregulatory Responses to Thermal and Chemical Stimulation and Inhibition of TRPV4 Channels in 60-Day-Old Adult Chickens

**FIGURE 4:**
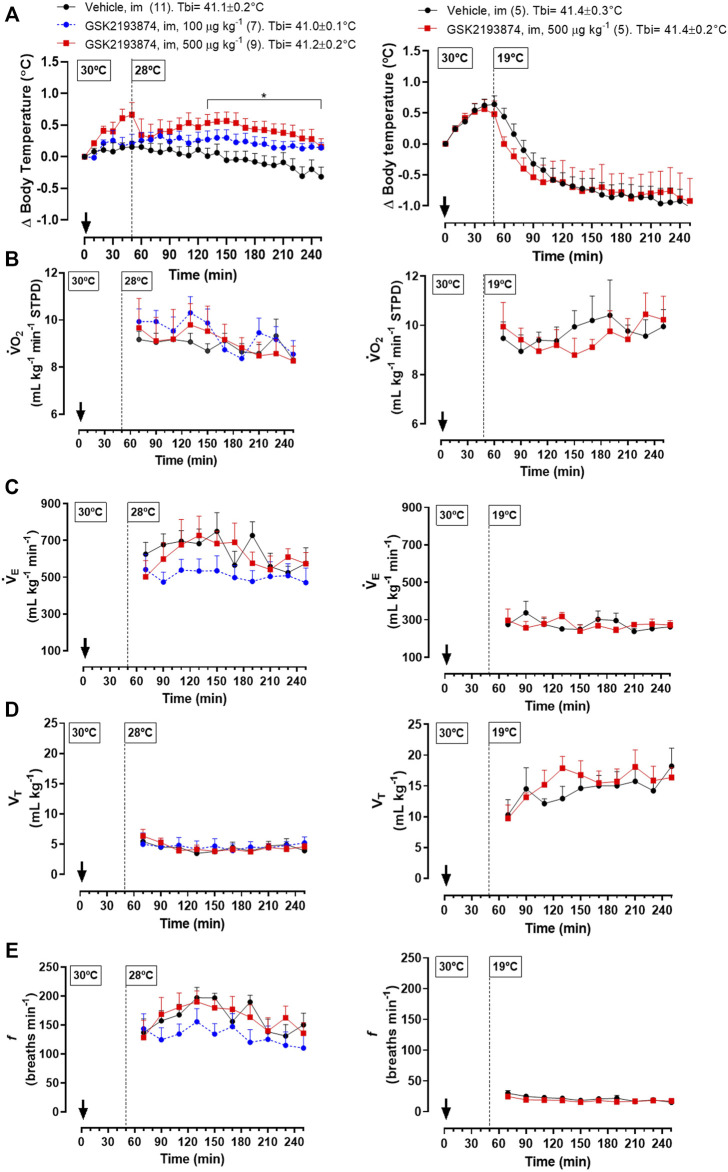
Effect of intramuscular injection (i.m.; 1 ml kg^−1^) of the TRPV4 antagonist, GSK2193874 (100 and 500 μg kg^−1^), or vehicle (1% DMSO) on body temperature (T_b_; **A**), oxygen consumption ( $\dot{V}$ O_2_; **B**), ventilation ( $\dot{V}$ _E_; **C**), tidal volume (V_T_; **D**) and breathing frequency (*f*; **E**) of 60-day-old chickens under warm condition (T_a_ = 28°C; graphs in the left). Effect of the i. m. injection of GSK2193874 (500 μg kg^−1^) or 1% DMSO (1 ml kg^−1^) on the same respective variables in 60-day-old chicken under mild cold condition (T_a_ = 19°C; graphs on the right). Tbi, initial body temperature. The arrow indicates the moment of injection. Number of animals in each group is shown in parentheses. All the animals were pre-exposed to a hot condition (30°C) for inducing cutaneous vasodilation and facilitate the distribution of drugs throughout the peripheral sites of receptors (Almeida et al., 2012). *p < 0.05, significant difference from the vehicle group at the same time point (two-way ANOVA; Sidak post-hoc test).

*Protocol 5*—Injection of 500 μg kg^−1^ (i.m.) of GSK 2193874 in adult chickens resulted in a higher T_b_ compared to the vehicle group at 28°C (Figure 4A left panel; 130–260 and 230 min; treatment effect: *p* = 0.01, F_(2, 24)_ = 5.13). A lower dose of the antagonist (100 μg kg^−1^) did not affect T_b_ significantly. No difference in $\dot{V}$ O_2_ (Figure 4B, left; treatment effect: *p* = 0.81, F_(2, 24)_ = 0.21), $\dot{V}$ _E_ (Figure 4C, left; treatment effect: *p* = 0.28, F_(2, 24)_ = 1.14), V_T_ (Figure 4D, left; treatment effect: *p* = 0.96, F_(2, 24)_ = 0.04) or *f* (Figure 4E, left; treatment effect: *p* = 0.26, F_(2, 24)_ = 1.44) was observed among the treatments in chickens at 28°C.

Different from the exposure to 28°C, chickens at 19°C and injected with the higher dose of GSK 2193874 (500 μg kg^−1^) did not differ in T_b_ from the vehicle group (Figure 4A, right panel; treatment effect: *p* = 0.90, F_(1,8)_ = 0.02). In this condition, the antagonist also did not affect the thermoeffectors $\dot{V}$ O_2_ (Figure 4B right; treatment effect: *p* = 0.82, F_(1,8)_ = 0.05), $\dot{V}$ _E_ (Figure 4C, right; treatment effect: *p* = 0.98, F_(1,8)_ < 0.01), V_T_ (Figure 4D, right; treatment effect: *p* = 0.60, F_(1,8)_ = 0.30) or *f* (Figure 4E, right; treatment effect: *p* = 0.33, F_(1,8)_ = 1.05).

**FIGURE 5:**
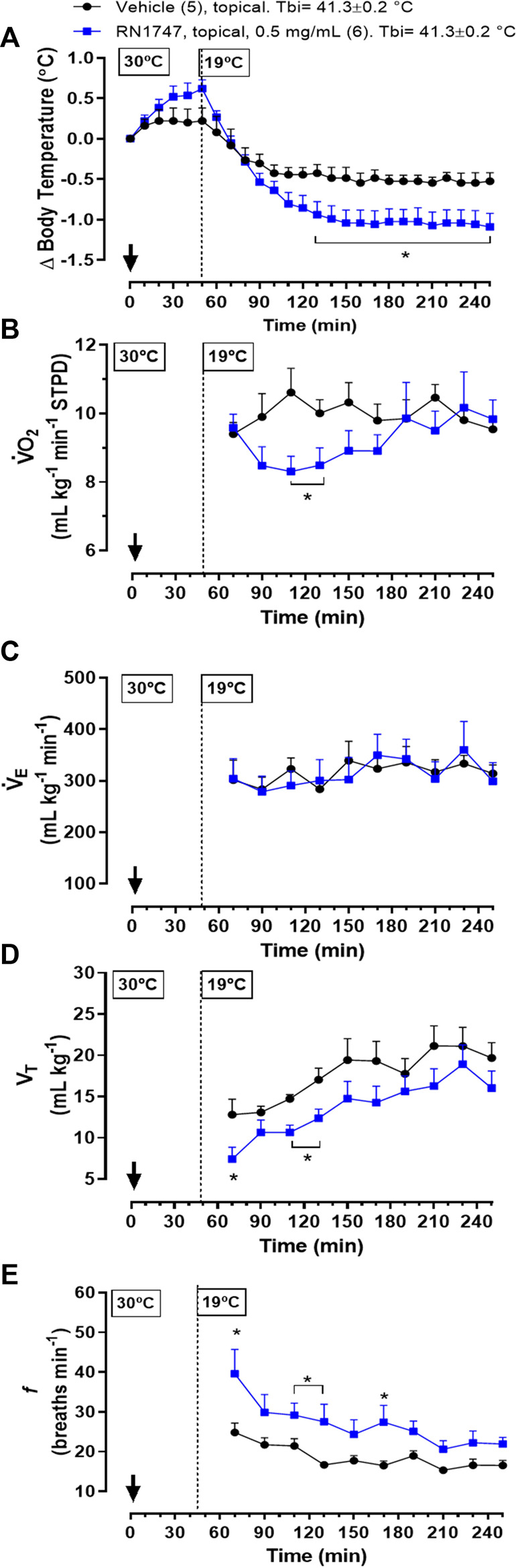
Effect of topical application (both dorsal and the ventral skin surfaces; 3.75 ml kg^−1^; ∼6 ml each side) of the TRPV4 agonist, RN1747 (0.5 mg ml^−1^), or vehicle (100% propylene glycol) on body temperature (T_b_; **A**), oxygen consumption ( $\dot{V}$ O_2_; **B**), ventilation ( $\dot{V}$ _E_; **C**), tidal volume (V_T_; **D**) and breathing frequency (*f*; **E**) in 60-day-old chickens under mild cold (T_a_ = 19°C). T_b_i, initial body temperature. The arrow indicates the moment of application. The number of animals in each group is shown in parentheses. All the animals were pre-exposed to a hot condition (30°C) for inducing cutaneous vasodilation and facilitate the distribution of drugs throughout the peripheral sites of receptors (Almeida et al., 2012). *p < 0.05, significant difference from the vehicle group at the same time point (two-way ANOVA; Sidak post-hoc test).

*Protocol 6*—Topical application of the TRPV4 agonist, RN1747, in 60-day-old chickens at 19°C resulted in a significant drop in T_b_ when compared to the vehicle group (Figure 5A; 130–250 min; treatment effect: *p* = 0.03, F_(1, 9)_ = 7.03). This T_b_ decrease in response to the agonist was preceded by a reduction in $\dot{V}$ O_2_ (Figure 5B; 110–130 min; treatment effect: *p* = 0.03, F_(1, 9)_ = 6.54). No difference in $\dot{V}$ _E_ was observed between groups (Figure 5C; treatment effect: *p* = 0.70, F_(1, 9)_ < 0.01). However, chickens treated with RN1747 had a lower V_T_ (treatment effect: *p* = 0.03, F_(1, 9)_ = 6.43) and a higher *f* (treatment effect: *p* = 0.03, F_(1, 9)_ = 6.20) than chickens injected with vehicle (Figures 5D,E), indicating activation of respiratory heat loss.

## 4 Discussion

**FIGURE 6:**
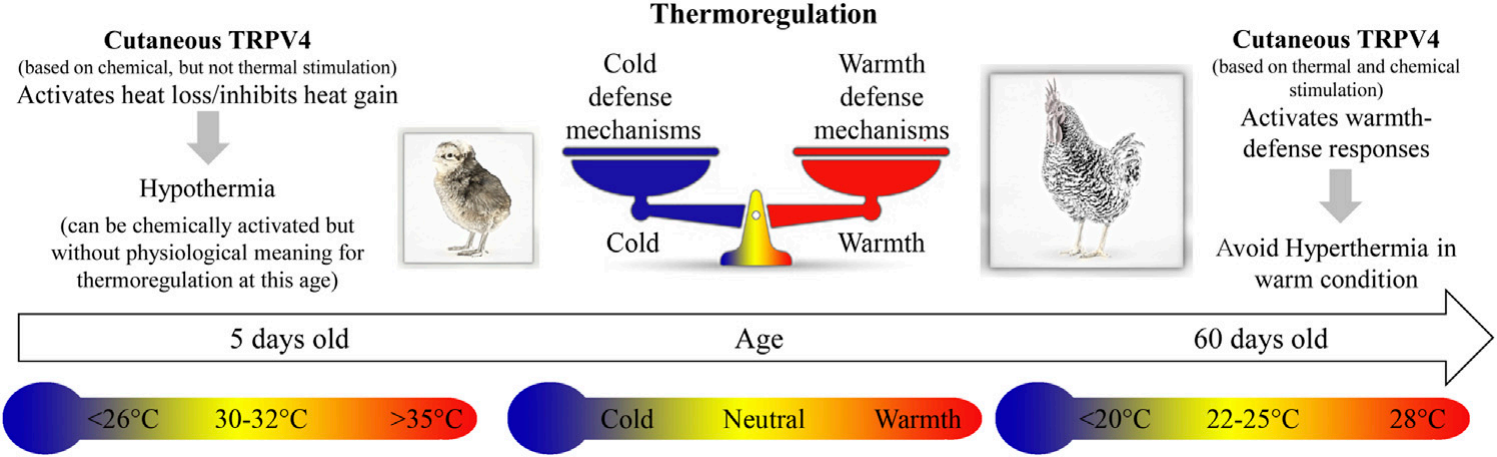
The cartoon illustrates the thermoregulatory effects of stimulation of cutaneous TRPV4 in chicken at different ages, 5 and 60 days old. In 5-day-old chicks, chemical stimulation of the TRPV4 receptors induces hypothermia, and specific antagonists inhibit this response. At this age, 30–32°C is considered thermoneutral, while 26°C or less and 35°C or more are below and above thermoneutrality, respectively. In 60-day-old chickens (adults), thermal stimulation (warm exposure; ∼28°C) of the TRPV4 receptors avoids hyperthermia. Moreover, at this age, chemical stimulation of TRPV4, even in cold condition (below 20°C), activates heat loss (warmth-defense) responses.

Our results show the first evidence of the presence of TRPV4 channels in the skin of the dorsal and ventral surfaces of the body, and their role in the activation of warmth-defense responses in birds. These thermosensors are functional in early life, despite having no physiological meaning for thermoregulation in chicks, which sense its temperature activation range (∼26–30°C) as cold to neutral (Figure 6).

Immunohistochemistry revealed the presence of TRPV4 channels spread throughout the dermis of chickens. At least in humans, these channels are highly diffuse in skin, including vascular endothelial cells, eccrine sweat gland secretory cells, and keratinocytes (Kida et al., 2012; Fusi et al., 2014; Olivan-Viguera et al., 2018). In rats, evidence exists for the presence of TRPV4 in keratinocytes (Guler et al., 2002; Wang and Siemens, 2015). In this case, it is suggested that some humoral pathways promote communication between the keratinocytes in the *epidermis* and the nerve endings in the dermis, which is essential for the thermal information to be sent to the brain (Wang and Siemens, 2015). In chickens, TRPV4 may reside in primary afferent sensory neurons and/or other cell types that are connected to nerve endings, which make possible the transmission of afferent signals for thermoeffector activation.

Chemical stimulation of cutaneous TRPV4 (topical application of a selective agonist) resulted in a decrease of T_b_ in 5-day-old chicks when allocated in both thermoneutral (31°C) and cold (26°C) conditions. The hypothermia observed during TRPV4 stimulation occurred together with the inhibition of huddling in response to cold (26°C), a behavioral heat conservation mechanism well known in birds, including chicken chicks (Kleiber and Winchester, 1933; McKechnie and Lovegrove, 2001; Gilbert et al., 2010; Amaral-Silva et al., 2022). Both the T_b_ decrease and the prevention of huddling behavior were inhibited by pretreatment with the TRPV4 antagonist (HC067047) at a dose that did not affect behavior when injected alone, highlighting the specificity of TRPV4 in this response. Interestingly, a similar pattern is observed in adult rats, i.e., the selection of colder T_a_s upon chemical activation of cutaneous TRPV4, and the inhibition of cold-seeking behavior by HC067047 following exposure to 28–31°C (Vizin et al., 2015).

Despite the pharmacological evidence that 5-day-old chicks have functional TRPV4 channels, and that HC06747 blunted the agonistic effect, the inhibition of TRPV4 channels alone at 31°C or 26°C did not increase T_b_, as observed in adult rats (Vizin et al., 2015; Scarpellini et al., 2019). This is probably because the TRPV4 thermosensors are not thermally activated at this age by either of the T_a_s used. Even though the chicks were exposed to T_a_s in the range that this thermoreceptor is shown to sense *in vivo* (Vizin et al., 2015), 31 and 26°C do not represent a warm T_a_ for the 5-day-old chicks. In fact, a T_a_ of 26°C triggers activation of thermogenesis instead of thermolysis in the first week of life in chicks (Amaral-Silva et al., 2021; Cristina-Silva et al., 2021; Amaral-Silva et al., 2022). Thus, these animals are not expressing any heat loss response, which would be inhibited by HC06747 in the case where TRPV4 was active, and T_b_ would be consequently increased by the lack of this response. We speculate that, at this age, TRPV4 channels may be chemically inhibited or present a very high activation threshold, becoming physiologically relevant only for older animals.

Later in life, when 28°C represents a supraneutral T_a_ for adult chickens, TRPV4 seems to be active and responsive, preventing hyperthermia in this condition. In support of this, when the adult chickens were exposed to mild cold (19°C), which does not activate TRPV4, the inhibition of these channels, using the same dose of antagonist as that which reduced T_b_ at 28°C, did not affect T_b_. The hyperthermic response to the TRPV4 antagonist at 28°C did not involve thermogenesis, considering that GSK2193874 did not change O_2_ consumption. Besides that, the thermal tachypnea (high *f* and low V_T_, known to provide respiratory heat loss; Hammel and Pierce, 1968; Richards, 1970; Mortola and Maskrey, 2011) was also not affected by the inhibition of TRPV4. In this case, if GSK2193874 only inhibited cutaneous vasodilation, an impairment of heat dissipation through the body-ambient thermal gradient (∼41.1–28°C) would be enough to cause the observed T_b_ increase (Richards, 1970; Wolfenson et al., 1981). This suggests that TRPV4 might maintain regular T_b_ in chickens exposed to mild suprathermoneutral T_a_s by peripheral vasomodulation, which may be enough for thermoregulation in the range of T_a_s sensed by TRPV4. Although it was not measured here, there is evidence that cutaneous vasodilation is activated by chemical stimulation of TRPV4 in adult rats (Vizin et al., 2015) and humans (Fujii et al., 2019). In the latter, however, that response is partially, but not completely, inhibited by local microinfusion of HC067047 or GSK2193874 (Fujii et al., 2021). This issue remains to be confirmed in birds. Another matter for future investigation is the possibility that TRPV4 channels are present in the brain of chickens. If this was the case, an influence of GSK2193874 inhibiting brain TRPV4 channels would be speculated. At least in rats, TRPV4 channels located in the hypothalamus region seem to play a role in thermolytic responses together with those channels present in the skin (Vizin et al., 2015; Scarpellini et al., 2019). Regarding chickens, although one cannot rule out a possible central action of GSK2193874, its efficiency in inhibiting cutaneous TRPV4 channels was confirmed by blockade of the hypothermia induced by topical application of RN1747 in chicks (Figure 3).

When TRPV4 was chemically stimulated at 19°C, adult chickens decreased T_b_, similarly to what was observed in chicks. This response in the adults relied on the activation of tachypnea, providing evaporative heat loss (Wolfenson et al., 1981; Arad and Marder, 1982), and also on metabolic rate inhibition, reducing heat gain (Amaral-Silva et al., 2021; Amaral-Silva et al., 2022). In this case, the pharmacological effect of the TRPV4 agonist may be more potent to stimulate the specific skin sensors for activating those thermoeffectors than stimulation by the warm condition at 28°C. A possibility exists that such a T_a_ is inside the common temperature range of activation of other temperature-sensitive receptors, for example TRPV3 (Luo and Hu, 2014; Wang and Siemens, 2015; Ishikawa et al., 2019), overlapping the influences of different thermoreceptors.

## 5 Conclusion

Our data indicate the presence of TRPV4 channels in the chicken’s dermis, which present a thermolytic role for T_b_ maintenance. Chicken TRPV4 thermosensors are functional in early life, when the range of TRPV4 activation (∼26–31°C) actually represents a cold to thermoneutral ambient condition. In this phase, these channels may remain suppressed, but become physiologically relevant only later in life, when the T_a_s they sense represent a warm condition for adult chickens and thermolysis is evoked to maintain T_b_. If there is a specific thermoeffector activated by TRPV4 channels in warm conditions, or if they induce small modulations of different thermoeffectors for maintaining T_b_, are still pending questions for birds.

## Data Availability

The raw data supporting the conclusion of this article will be made available by the authors, without undue reservation.

## Abbreviations

A, wave amplitude; CNS, central nervous system; *f*, respiratory rate; FeO_2_, excurrent fractional concentration of oxygen; FiO_2_, incurrent fractional concentration of oxygen; FRe, excurrent flow rate; P_B_, barometric pressure; Pb_H2O_, water vapor pressure of the air inside the animal’s body; P_cal_, calibration pressure; Pc_H2O,_ water vapor pressure in the chamber; POA, preoptic area of the anterior hypothalamus; RQ, respiratory quotient; STPD, standard conditions of temperature, pressure and dry air; T_a_, ambient temperature; T_b_, body temperature; T_ch_, temperature inside the respirometry chamber; TRPA, transient receptor potential ankyrin; TRPC, transient receptor protein channel; TRPM, transient receptor potential cation channel subfamily M (melastatin); TRPV, transient receptor potential vanilloid; Ts, surface temperature; V_cal_, calibration volume; $\dot{V}$ _E_, pulmonary ventilation; $\dot{V}$ O_2_, oxygen consumption; V_T_, tidal volume.
